# Monitoring System for Railway Infrastructure Elements Based on Thermal Imaging Analysis

**DOI:** 10.3390/s21113819

**Published:** 2021-05-31

**Authors:** Krzysztof Stypułkowski, Paweł Gołda, Konrad Lewczuk, Justyna Tomaszewska

**Affiliations:** 1 Faculty of Transport, Warsaw University of Technology, Koszykowa 75, 00-662 Warsaw, Poland; krzysztof.stypulkowski@pw.edu.pl; 2 Air Force Institute of Technology, Księcia Bolesława 6, 01-494 Warsaw, Poland; pawel.Golda@itwl.pl; 3 Faculty of Aviation, Military University of Aviation, Dywizjonu 303 35, 08-521 Dęblin, Poland; j.tomaszewska@law.mil.pl

**Keywords:** railway safety, information system, thermal imaging, track condition, electric heating of turnouts

## Abstract

The safety and reliability of railway transport requires new solutions for monitoring and quick identification of faults in the railway infrastructure. Electric heating devices (EORs) are the crucial element of turnouts. EORs ensure heating during low temperature periods when ice or snow can lock the turnout device. Thermal imaging is a response to the need for an EOR inspection tool. After processing, a thermogram is a great support for the manual inspection of an EOR, or the thermogram can be the input for a machine learning algorithm. In this article, the authors review the literature in terms of thermographic analysis and its applications for detecting railroad damage, analysing images through machine learning, and improving railway traffic safety. The EOR device, its components, and technical parameters are discussed, as well as inspection and maintenance requirements. On this base, the authors present the concept of using thermographic imaging to detect EOR failures and malfunctions using a practical example, as well as the concept of using machine learning mechanisms to automatically analyse thermograms. The authors show that the proposed method of analysis can be an effective tool for examining EOR status and that it can be included in the official EOR inspection calendar.

## 1. Introduction

Recent years have shown that globalization and chasing the latest ecological and technological trends have found railways playing a significant role in transportation worldwide. This enables national railway companies to justify modernization that increases safety and speed. The railway transport processes pose a number of difficulties for carriers, e.g., related to meteorological conditions, which are of great importance on distant routes and in different latitudes. These difficulties can be overcome by new railway technologies related to the maintenance and preservation of jointless track [1,2], traction energy consumption [3], lower noise emissions [4], and autonomation of rail transport [5]. However, to ensure efficient travel in difficult weather conditions, railway companies have introduced turnouts equipped with an electric turnout heating system (EOR) to counteract the effects of low temperatures and icing. Such solutions are intended to adapt the lines to the faster railway traffic and to increase the automation of railway infrastructure. The actual technologies allow automatic switching on the heating devices depending on the temperature changes, which overcomes icing problems. However, the control and inspection procedure of EORs requires stopping the railway traffic and conducting time-consuming tests.

An EOR allows the switch to be freely and safely repositioned to adapt its setting to the current traffic situation. Railroad turnouts are complex technical structures. Turnout elements such as resistors at the moveable section of switch rails, crossings equipped with a moveable point, channels under the closure, setting closures, and others [6] require winter heating. An EOR prevents turnouts from freezing and facilitates snow and ice removal, increasing the reliability of rail transport. The key issue is to organize and supervise the performance of the railway infrastructure monitoring system in accordance with the Safety Management System (SMS) that is in force. Based on the definition in European Parliament Directive 2016/798, SMS means the organization, measures, and procedures adopted by an infrastructure administrator or a railway company to ensure the safe management of the operation. In many cases it can be ensured by combination of thermal imaging methods for quick testing and artificial intelligence for image analysis for monitoring and predicting the damage to the devices that support the traffic of rolling stock and railway rails.

Early detection of rail and turnout element failures is an indispensable element of a railway traffic safety system. The introduction of innovative solutions such as thermal imaging in rail transport can be modelled on solutions in the area of air transport [7,8] or on methods and tools supporting the reduction of environmental destruction and safety of transport [9,10]. The aim of this paper is to present a method based on thermal imaging of infrastructure elements that can be implemented in a railway infrastructure monitoring system. The computer solution proposed by the authors for image analysis is to be equipped in the next stage with artificial intelligence methods to recognize thermographic patterns and to search for defects.

The rest of this article is divided into four parts. In the first section, a critical analysis of the literature on rail defect detection techniques for railway systems and machine learning-based modelling used for inspection is conducted. In the next section, the characteristics of the investigated object are presented, indicating the essence of the operation of trackside EOR devices and their analysis using a thermal imaging camera. The EOR track devices and parameters used to perform image analysis and detection using thermal imaging are described. The third section is a description of an information system supporting safety management based on image analysis. The last section of the article presents and discusses the results of the research. In the Conclusions, the authors point out the premises for expanding the area of infrastructure covered by the proposed system, a system that can significantly increase the level of safety of the infrastructure in use through faster diagnosis of damaged elements and indication of emergency states.

## 2. Literature Analysis

### 2.1. Rail Defects Detection

Safety is a key issue for modern railways. One of the most common types of adverse events is train derailment due to rail deformation [11]. A lot of research is currently in progress, both on the quality of the materials used as well as on the construction technique of rail systems, in order to increase safety. One way to increase safety is to improve the quality of steel used in the production of rails. For example, harder steels lead to longer line life and reduce the likelihood of rail defects. However, as traffic increases, rail failures remain inevitable.

Early detection of rail damage is necessary to repair the tracks before defects cause serious problems such as derailments. Railway track damage is mainly detected using an instrumented car as a monitoring vehicle [12,13]. Instrumented vehicles carry various sensors and devices including cameras, accelerometers on the car body, acoustic sensors, ultrasonic wave sensors, global navigation satellite systems (GNSS), linear potentiometers, strain gauges on the top chord, etc. [14]. A rail defect detection system consists not only of an instrumented vehicle with a set of sensors but also a data acquisition system, a data analysis tool, and data processing algorithms. Railway track inspection uses non-destructive techniques [15,16] which can be divided into vision-based methods such as laser scanners [17] and video image processing [18], as well as displacement-based methods such as acceleration measurement [19], which measure the vertical movement of vehicles to detect damage to rails and other track components. Thermal imaging cameras are also of great importance in diagnosing rail defects. Thermal imaging cameras capture infrared radiation emitted by objects whose temperature is above absolute zero. This type of camera was originally developed as a night vision and observation tool for the army. The use of thermal imaging systems for rail condition monitoring eliminates illumination-related problems that occur with normal grayscale cameras and RGB (red, green, blue) cameras. The authors of [20,21,22] present the results of thermal calculations, the important part of which is the analysis of the energy efficiency of electric heating of railway turnouts for which the use of electric heaters is considered. Attention has been paid to the contribution of particular heat exchange mechanisms in the course of the heating phenomenon in the turnout working space.

An interesting take on the use of thermal imaging to detect irregularities in rails can be found in the paper [23] where an experiment was performed on real objects. In this study, a method was sought to detect the phenomenon of “rolling”, which is impossible to detect by profile inspection or visual inspection. The authors prove that the use of thermal imaging allows the detection of this phenomenon despite the fact that the only methods for detecting this type of defect known so far are complex inspection systems based on eddy currents. On the other hand, Oswald-Tranta 2018, in his work [24] presented the use of thermal imaging to investigate metal damage, where the tested sample is heated with a short induced impulse of electric current and a thermal imaging camera records—during and after the heating impulse—the temperature distribution on the surface. Converting the temporal development of temperature for each pixel into information not only provides highly reliable detection of possible cracks but also provides an estimate of their depth. Finite element simulations were performed to investigate how the phase contrast depended on parameters such as excitation frequency, impulse duration, material parameters, crack depth, and crack angle. Based on these results, generalized phase difference dependence functions were derived for all these parameters. The author hypothesizes that these functions can serve as guidelines for optimizing the measurement parameters for a given material so that cracks can be detected and their depth estimated.

Netzelmann et al. 2016, [25] presented a comprehensive survey on induction thermograms used for surface defect detection in forged elements and a method of crack detection in railway components such as rails and wheels. They propose using a test car moving at a speed of up to 15 km/h. The authors note that in recent years thermography became standardized and that this will soon lead to new standards on active thermography and flash excited and induction thermography. Publication [26] presents an all-purpose crack detection algorithm for flying spot thermography. The algorithm does not need the initial information on the experimental setup. The spatial derivative is calculated for frames of the sequence and, secondly, the resulting data set is sorted pixel-wise along the time axis.

In [20,21,27] the author rightly notes that electric heating of turnouts is a significant technical and economic problem. For these reasons, there is a need for research on optimising the rail turnout heating system. In his research, the author presents the results of a numerical analysis of turnout heating systems using the classical method and heaters thermally isolated from the rail foot. He performed 2D and 3D analysis of the systems under consideration, on the basis of which he demonstrated the validity of using the 2D model for calculations. Numerical calculations of the turnout heating process were performed in the ANSYS software. In this work, the author referred to the energy efficiency of electric turnout heating equipment. A comparative analysis of energy losses during heating of railway turnouts used two different methods. The turnout heating analysis was performed in the ANSYS software. An analysis of resistive and inductive heating of railway turnouts was presented in [22]. In these studies, appropriate thermograms were included, which showed the temperature distribution on the surface of the device tested.

The need for conducting diagnostics of railway turnouts with special attention to the problem of image diagnostics is noted by [28]. The author presents the principles of correct photography of the tested surface element, which, apart from the verbal record, is particularly useful in the diagnostic process and the evaluation of changes occurring over time. The topic is not directly related to thermal imaging, but the issue of archiving the study results, including thermograms, is important in relation to the proposed IT solution. The author correctly states that a railway turnout is one of the more complicated parts of a railway road. During its operation, special attention should be paid to problems related to its proper maintenance. To this end, ongoing and periodic inspections should be conducted to allow for early detection and remedy of defects, thereby extending the service life of railway turnouts. During the inspections of turnout elements, especially visual, detected defects should be archived in the form of photographs or thermograms. The documentation produced in this way can be used in subsequent periodic reviews to analyse the changes taking place.

The administrator of the national network of railway lines in the studies, referred to as instructions [6,29] defines the guidelines for the design of electric heating devices of Iet-5 turnouts and introduces the instructions for operation and maintenance of devices of electric heating of Iet-1 turnouts in publication [30], normative document 01-8/ET/2008. The aforementioned studies constitute annexes to the ordinances of the Management Board of PKP Polskie Linie Kolejowe S.A. (Polish National Railways) These compulsory instructions must be applied for designing and comprehensively upgrading electric turnout heating devices within the territory of PKP Polskie Linie Kolejowe S.A. Instructions, guidelines, normative documents are intended for:

- Designers of EOR devices;
- Manufacturers of railway turnouts and manufacturers of EOR devices;
- Contractors implementing the installation of EOR devices;
- Employees of supervision and operation of electric turnout heating devices in PKP Polskie Linie Kolejowe S.A.

### 2.2. Machine Learning-Based Modelling for Railway Inspection

One of the important tasks for the administrator of railway infrastructure responsible for traffic safety is to develop comprehensive tools for rail and equipment damage detection which use the raw data but which do not apply complicated engineering of metal behaviour under the influence of external factors. In this aspect, data analysis based on machine learning (ML) methods has great application potential.

Data analysis based on machine learning has been used in railways for years. To date, however, most of the data analysed have come from studies based on displacement testing and analysis. Car body acceleration signals, i.e., six degrees of freedom (DOF) or six modalities of vehicle body motion, i.e., roll, pitch, yaw, lateral, vertical, and longitudinal, are investigated using machine learning techniques. An example of the use of machine learning is provided in [31]. Authors use ten popular regression algorithms to predict vehicle motion using the vertical acceleration of the car body. The authors evaluated the performance of different models based on statistical hypothesis analysis. Based on the experimental test results, it has been shown that the application of machine learning techniques is effective and allows prediction of the vertical motion characteristics of the front and rear of the car body mass with negligible errors.

Machine learning is data analysis, one of the basic elements of which is classification, which consists of assigning the analysed object or phenomenon to one of a set of defined classes. Defining the classes is usually one of the first tasks of phenomenon analyses. In all cases for which there are no predefined groups, machine learning techniques are used to define them [32]. ML techniques include an extensive class of algorithms ranging from decision trees and genetic algorithms to metric techniques such as k-NN, SVM, statistical methods, Bayesian networks, and artificial neural networks. These methods are intended to support the construction of an intelligent algorithm or system that allows for predicting the technical condition of a given object based on an assessment of the current situation of the object.

The application of artificial neural networks in the analysis of the condition of railway infrastructure elements seems to be particularly promising. The authors of [33] presented rail track inspection methods based on feature identification and a deep neural network method using acceleration data. The deep learning-based method complements the classification-based detection method. The advantages of using deep neural networks are mainly the significant reduction in data pre-processing time for feature extraction. Moreover, this method affords the possibility of detecting anomalies on the left or right rail using one global model. As research indicates, the use of neural network-based methods eliminates the problem of data disturbance. In the researches [34,35] authors indicate that deep neural networks are applicable wherever it is necessary to replace the work usually done by humans with work done by computers without any loss of efficiency. Non-railway application areas of deep neural networks may also be relevant to railway applications. In [36] the author presents the feasibility of using co-evolutionary neural networks for the detection of pulmonary nodules. The aim of this study was to improve the performance of a deep-learning-based CAD system for automatic detection of pulmonary nodules. The authors achieved comparable performance among CAD systems evaluated in the LIDC-IDRI database. The combined results from the three planes showed better performance than the result from any single plane, indicating that the three planes may provide complementary information for the detection of pulmonary nodules.

The literature on thermography used for the detection of faults and damage in railway infrastructure presents examples of methods and case studies. Thermography, as a non-destructive tool, is especially valuable for railway infrastructure monitoring. This problem is a broad problem which can be discussed from many perspectives. Table 1 presents topics that arise in the literature on the problem.

Although there are well explored areas of research, it is still possible to develop new methods for new purposes. The testing of EOR devices is not commonly present in the literature and represents a relatively new area in this field. In addition, the potential use of machine learning for EOR thermograph imaging creates a new research niche in thermography. This niche is explored in this article.

## 3. Characteristics of the Tested Object

### 3.1. EOR Trackside Devices Selected for Analysis and Image Detection Method Using Thermal Imaging

**Figure 1:**
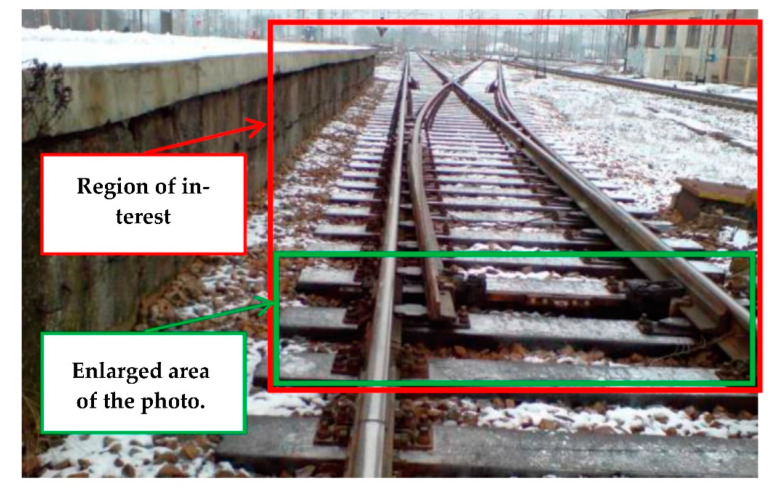
Photo showing a fragment of the track layout (source: authors).

**Figure 2:**
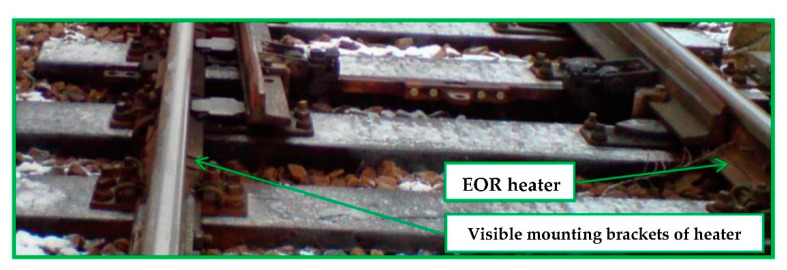
Enlarged area of the photo showing a fragment of the turnout track layout (source: authors).

**Figure 3:**
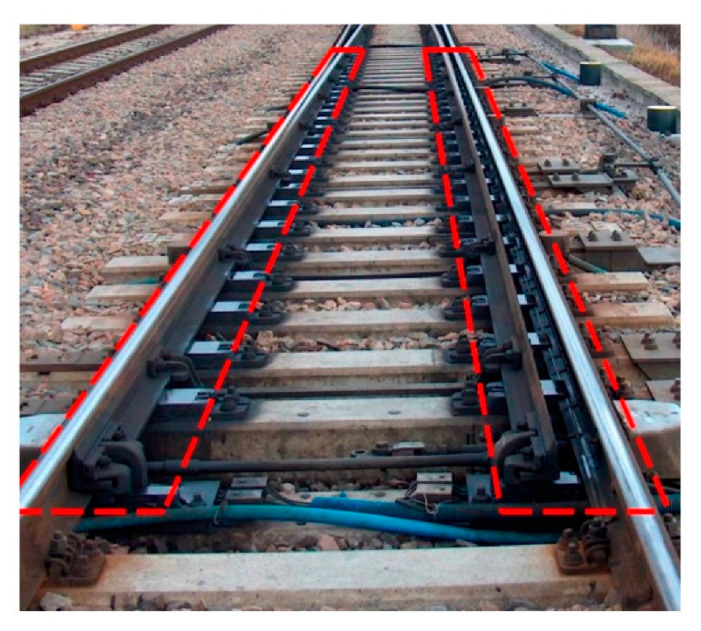
Common turnout equipped with an EOR device: heated space between the resistor and the turnout switch rail (source: authors).

Figure 1 and Figure 2 show selected exemplary fragments of a track layout with marked analysis areas. The basic component of EOR devices is the resistor—the stationary part of the track switch that is particularly vulnerable to icing and snow cover. The most critical area is the space between the resistor itself and the turnout switch rail, which requires heating to remove snow and ice. This is done using resistor heaters attached to the resistor foot [6]. The heated space between the resistor and the turnout switch rail is presented in Figure 3.

Initially, the heating process is related to the allocation of thermal energy that increases the temperature of the wire. A small amount of heat is also transferred to the environment. Over time, the wire heats up, implying an increase in the energy given off to the environment in the form of heat. After a while, there is a balance between the heat produced and that given off to the environment. It is a thermally steady state that can be described by a steady-state temperature. The steady-state temperatures of the EOR heaters are shown in Table 2.

Resistor heaters should be constructed with the following basic components:

- A heater rod is a metal jacket of the heater with a flat-oval cross-section, resistant to weather conditions, inside which a heating coil electrically isolated from the jacket is placed.
- A 3-wire copper power cable in flexible insulation (sheath) with increased resistance to mechanical damage is suitable for operation in places exposed to grease and temperatures from −40 °C to +70 °C. The wires of the power supply cable should have insulation colour in accordance with PN-EN 60068-2-6:2002: black or brown for the power wire; blue for the neutral wire, yellow and green for the protection wire.
- A hermetic coupler connects the power supply cable with the heater rod.

The heater rod should be free of twists and bends. Its surface should be smooth and even, without protrusions or depressions and without mechanical damage. The dimensions of resistor heaters should be in accordance with Table 2.

The dimensions of the bar section must be as follows: width = 12^+0.5^_-1_ mm and height = 6^+0.5^_-1_ mm or, alternatively, width = 13^+0.5^_-1_ mm and height = 5.5^+0.5^_-1_. The length of the supply cable (without the coupler) in the resistor heaters should be: version 1 = 4200 mm ± 200 mm, version 2 = 1200^+200^_-100_ mm. The cross-section of power supply cable wires should be 1.5 mm^2^. The recommended temperature thresholds for heating the turnout resistors are defined in [29] Table 2.

The EOR devices located in the track layout of the Warszawa Zachodnia station under normal atmospheric conditions were selected for the tests. It was determined that the average temperature of the heating part of the tested heater supplied with 230 V AC after 60 min of heating should be above 160 °C with the heat recovery [30].

The procedure for testing the heating parts of an EOR device involves a series of formally described steps [30]. A measured temperature at any points of the heating part of the heater rod (jacket) or on the surface of the heating panel may not differ by more than ±50 °C, and the maximum temperature difference between any points of the heating part may not exceed 70 °C. The temperature of the heating part of the heater jacket is measured at six points by means of temperature sensors or a temperature probe. At this stage, it is proposed that testing include thermal visual inspection. After 60 min of heating, when supplied with the rated voltage, temperatures should be read, and the average temperature of the heating part of the heater rod should be calculated. The test result should be considered positive if all the requirements according to item 5.5.1. of the normative document 01-08/ET/2028 *Heaters for Electrical Heating of Turnouts* [30] are fulfilled. The steady-state temperature of the heater, measured at any point of the heating part of the heater, should be in accordance with Table 2 for a resistor heater below 400 °C with heat recovery supplied at AC 253 V. After checking the average temperature of the heater jacket, and after the heater has cooled down to ambient temperature, a voltage higher by 10% than the rated voltage should be energized. The heater should be heated until the temperature is steady, then the temperature should be measured at five points of the heating section. The test result should be considered positive if all the requirements according to item 5.5.2 of the normative document 01-08/ET/2028 *Heaters for Electrical Heating of Turnouts* [30] are fulfilled.

### 3.2. Track-Side EOR Equipment Maintenance vs. Thermal Imaging Camera

EOR devices, as defined in Section 3.1, have well-defined operation and maintenance guidelines and require continuous condition monitoring. The permission to operate requires a positive result of the periodic inspection, an update of technical documentation, the control of alarm occurrence, as well as training of the employees on the system operation [29]. The basis for periodic inspections of EOR devices is a list of turnouts that will be heated during the heating season by the end of June. The inspections must be completed by the end of October each year. Instruction [39] defines the way to secure places of inspection.

EOR maintenance involves a series of standard activities:

(a) Determining the extent of wear or damage to EOR devices components;
(b) Carrying out necessary repairs of the devices or replacement of components;
(c) Carrying out measurements of technical parameters (in a one-year cycle—testing of anti-shock protection efficiency of EOR devices, testing of the state of insulation of transformers and EOR transformer boxes, in a five-year cycle—measurements as in the case of the one-year cycle and testing of the state of insulation of power, control and signalling cables, testing of earthing);
(d) Performance of maintenance operations;
(e) Painting, if necessary, identification markings in accordance with the guidelines applicable in national carrier (here PKP Polskie Linie Kolejowe S.A.).

The EOR device maintenance activities include regular service (visual inspection, maintenance, troubleshooting, adjustment of weather sensors [29]), emergency service, periodic inspections, and scheduled and ongoing repairs. Maintenance and periodic inspection procedures should be recorded in the *Device (EOR) Record Book—Part B*. In case of abnormal and insufficient heating, the event should be recorded in the *Device (EOR) Record Book—Part C*, and the plant dispatcher and device maintenance (EOR) operator should be notified [29]. Taking devices out of service should be recorded in the *Device (EOR) Record Book—Part A*.

Visual inspection is for the assessment of the technical condition of the visible components of EOR devices and can be done when the power is on during the heating season. Any defects should be rectified immediately if they threaten the safety of the railway, the environment, and the operation crew [29]. This part of maintenance is a time-consuming operation in which thermal imaging can present an advantage. Maintenance of EOR devices also includes cleaning, lubrication, touch-up of paint, and functional checking, which can be also subjected to thermograph analysis [29].

The inspection is confirmed through the *Report of periodic inspection of EOR devices* [29]. In-service measurements are made during periodic inspections. These include power and control lines, switchboards (EOR), circuits rated at 1 kV, transformers (EOR), automatic control machines, control panels, and heaters (EOR) [29]. Operational measurements of the EOR system are shown in Table 3.

In-service measurements largely involve checking the operation of shock protection measures. Effectiveness of this protection must be checked in EOR switchgears, circuits with rated voltage of 1 kV, circuits of automatic control machines with metal casing, and outlet circuits of control boards in signal boxes. The inspection is additionally required after repairs and replacement of components affecting the protection against electric shock. The confirmation of execution of operation measurements is the *Insulation Resistance Test Report* and *Report for Testing the Effectiveness of Electric Shock Protection of EOR devices* [29].

A distinction is made between emergency, current (ongoing), and scheduled repairs to EOR devices. Emergency repair is the repair of faults and defects that have developed on EOR devices. Ongoing repair is done during a periodic inspection before the heating season begins. Scheduled repair is the restoration of the original technical parameters according to a predetermined repair plan. Examples of planned repairs include ageing of wires and cables, corrosion of metal components, etc. In all cases a final inspection is required.

The inspection of devices (EOR) is conducted by an employee of the infrastructure operator with the required building license. The frequency of inspection performance is laid down in Article 62 of the *Building Law* (legislation system of Poland) and amounts to one year. It includes checking the technical condition of devices, fire protection efficiency reports, insulation resistance reports, and the repair quality reports. The person carrying out the inspection is obliged to prepare the inspection report, to give an assessment of the technical condition, and to make recommendations [30]. Documentation allows the planning of replacements and repairs and must be constantly updated. Thermal images with the report from analyses could be used as a part of documentation for further comparison.

In-service measurements are not currently supported by thermal imaging, especially when combined with machine learning for device condition recognition. The use of such methods will improve maintenance processes and enhance the preparation of technical documentation of EOR devices. Performing tests with a thermal imaging camera will improve the performance of selected operational measurements listed in Table 3 and will directly affect the safety of the EOR system. It is recommended that consideration be given to incorporating thermal imaging measurements into in-service measurements of devices.

## 4. Thermographic Testing of EOR Element

### 4.1. Meteorological Conditions

**Figure 4:**
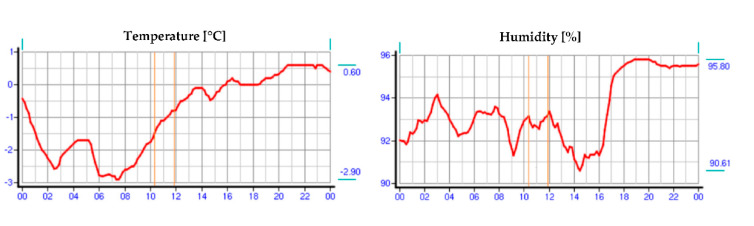
Temperature and humidity range on the day of the study (source: authors).

**Figure 5:**
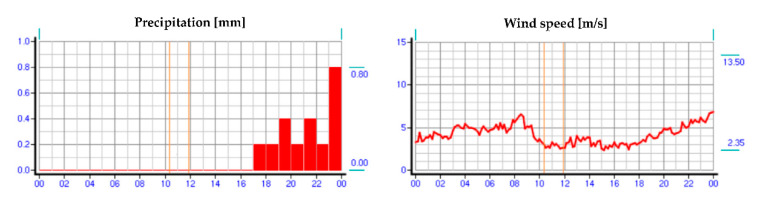
Precipitation and wind speed values on the day of the study (source: authors).

**Figure 6:**
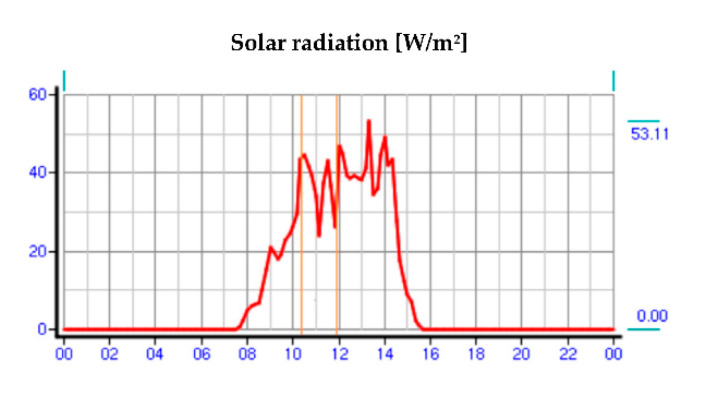
Parameters describing solar radiation on the day of the study (source: authors).

On the day of the study, meteorological data were collected from a weather station located on the border of Warsaw and Reguły. The atmospheric conditions prevailing on that day during the hours of the turnout inspection are shown in Figure 4, Figure 5 and Figure 6, respectively, where the hours of the measurements are marked.

**Figure 7:**
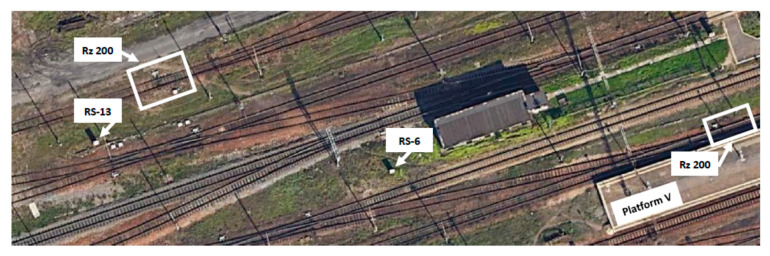
Satellite photo of a part of the track layout of the Warszawa Zachodnia station with marked turnouts and the apparatus and supply cabinets assigned to them (source: authors).

The atmospheric conditions were favourable for performing measurements; the measurement area was covered with snow, allowing observation and additional visual inspection of the active EOR system. The visual inspection was carried out for the common turnouts Rz no. 113 and Rz no. 200 in Warszawa Zachodnia station in Warsaw, Poland (Figure 7).

### 4.2. Thermovision Imaging od EOR Device

**Figure 8:**
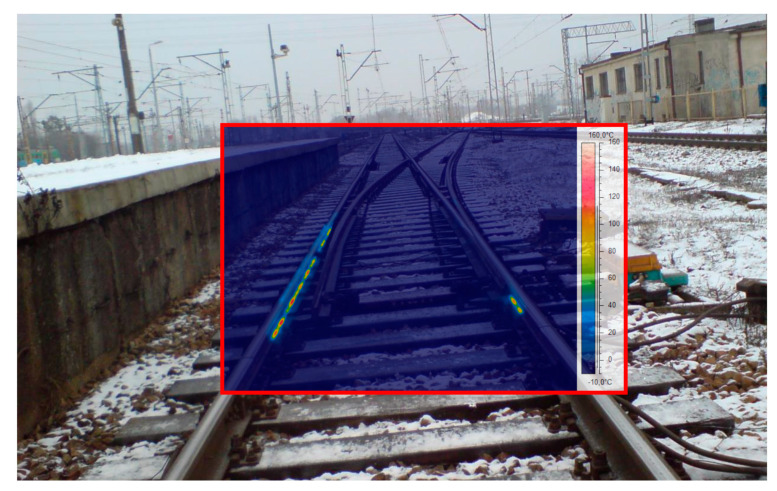
Photo of a fragment of a Rz 200 turnout in the track layout near Warsaw (source: authors).

Thermovision is a research method that tracks the processes associated with changes in emissivity or temperature over time or with the differentiation of thermal images of individual objects (as in the case of EOR). A camera with a non-cooled SC 660 detector by FLIR was used for the tests. It was mounted on a tripod at a height of 1.3 m placed in the track axis at a distance of 3 m to 10 m from the object. Thermograms were recorded in automatic mode with the emissivity of 0.95. The span and temperature level were corrected for selected thermograms. The emissivity was corrected assuming the emissivity of the steel (rusty) of 0.69. A thermal imaging camera can take a picture in a visible range and in the infrared range (Figure 8)—thermogram.

**Figure 9:**
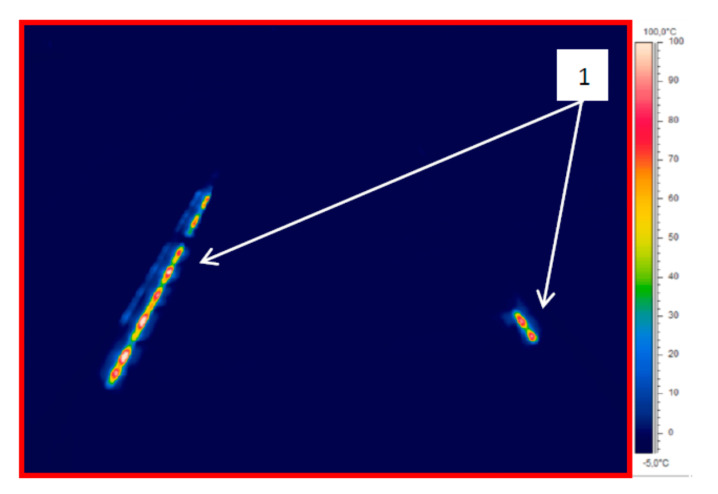
Thermogram IR_0145—rain900 colour palette (source: authors).

**Figure 10:**
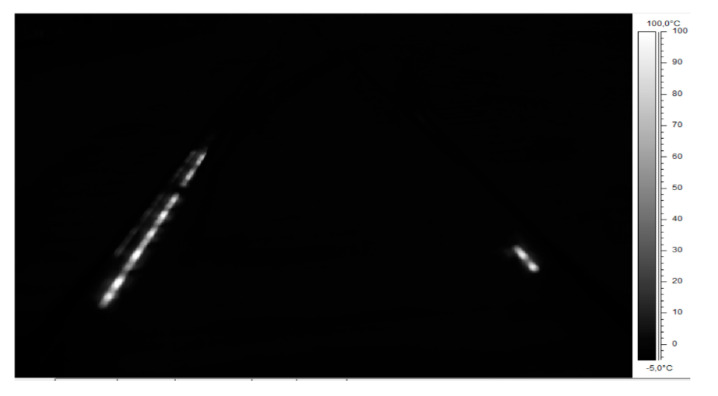
Thermogram IR_0145—grey colour palette (source: authors).

**Figure 11:**
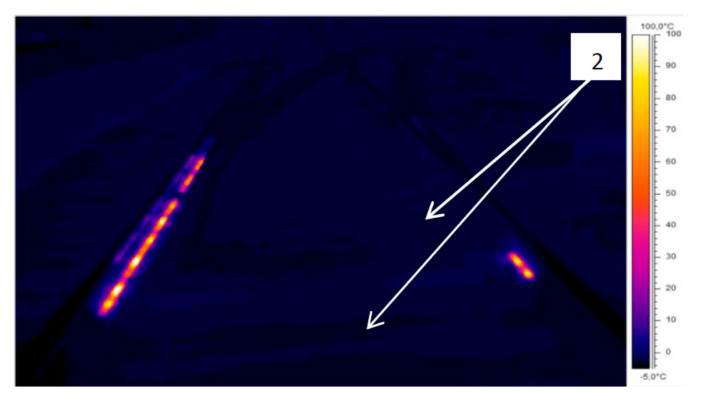
Thermogram IR_0145—iron colour palette (source: authors).

Figure 9, Figure 10 and Figure 11 show thermogram no. IR_0145 of the EOR device presented in Figure 8 using different colour palettes with temperature span from −5 °C to 100 °C. In Figure 9, reference 1 marks the resistor heaters visible in the infrared; the other elements of the infrastructure are not visible for the colour palette and temperature span adopted this way.

Figure 10 presents thermogram IR_0145 in grey scale, and Figure 11 presents it using the iron colour palette. Image presented in Figure 11 reveals the track system infrastructure (reference 2), together with active EOR system heaters, rails, sleepers, ballast, and the outline of the platform side wall. These characteristics are not visible in Figure 9 and Figure 10 with different colour palettes.

**Figure 12:**
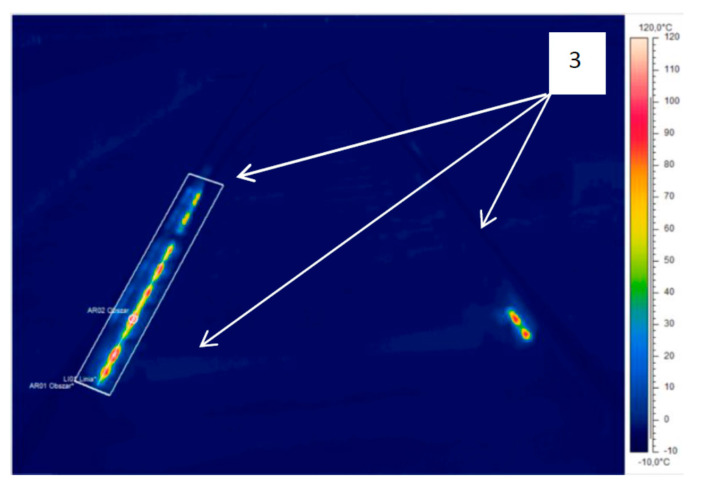
Thermogram IR_0145, rain900 colour palette, span correction from −10 °C to 120 °C (source: authors).

**Figure 13:**
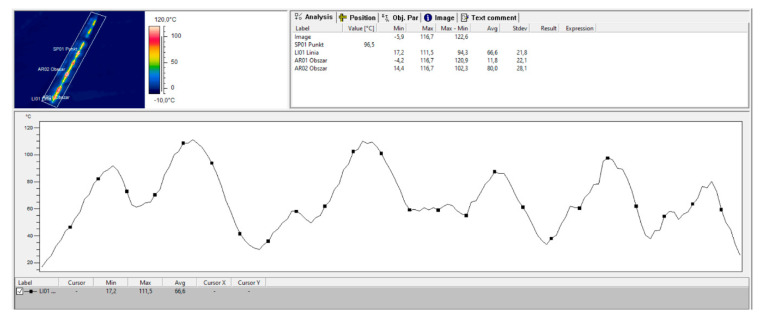
Thermogram IR_0145, rain900 colour palette, the temperature profile for line L01 tool is shown (source: authors).

Figure 12 and Figure 13 still refer to thermogram IR_0145, but the temperature span was changed in the imaging range, taking this parameter from −10 °C to 120 °C. The thermogram shown in Figure 12 contains visible elements of the track system infrastructure of the EOR system, marked by reference 3. The increase in the detection of resistor heaters is affected by the rain900 colour palette and the wider temperature range, which strengthens the thermal contrast.

Figure 13 shows the temperature profile for the L01 line tool dedicated to the thermal imaging camera, allowing the analysis of the temperature distribution in the heater alignment.

**Figure 14:**
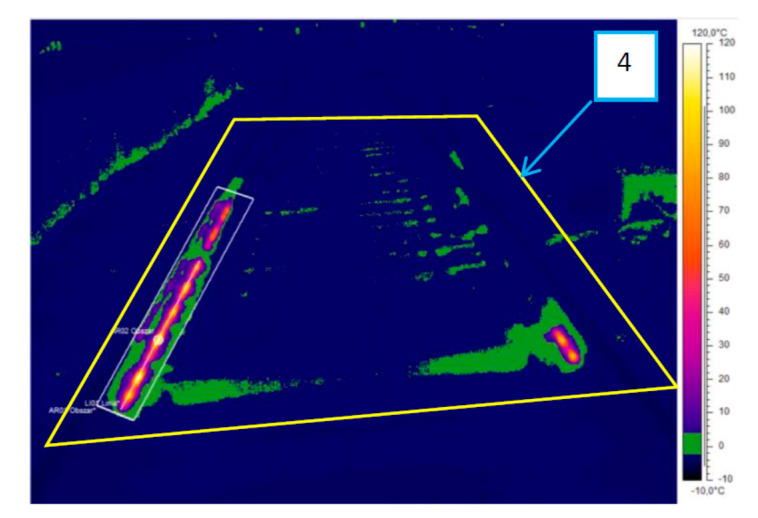
Thermogram IR_0145, iron colour palette, the isotherm tool is defined, and the area of effective influence of EOR devices is adopted (source: authors).

**Figure 15:**
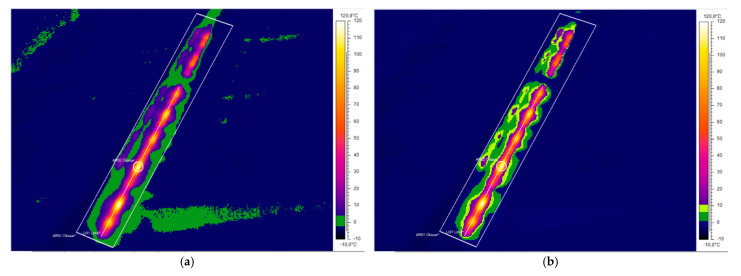
Thermographs: (**a**) zoomed region of interest IR_0145, iron colour palette, isotherm tool defined, (**b**) the two levels of the isotherm tool span.

A properly configured isotherm tool with applied image processing and analysis tools can be used for estimation of the energy efficiency of the ice-melting. Reference 4 in Figure 14 marks the area of the effective influence of the analysed turnout electric heating devices, the zoomed part of which can also be seen in Figure 15.

Thermographs obtained by the proposed image transformation with the magnification tool, temperature span adjustment, and isotherm tool can be used for manual inspection of EOR devices. The automatic inspection and forecasting of the time of wear require machine learning for making decisions on the need for manual inspection.

### 4.3. Digital Representation of Thermograph Imaging

By choosing the right colour palette, temperature span, and isotherm, the imaging can be adjusted to the need for manual inspection but also for the automated process. This approach to analysis and interpretation is applied in the software that is created, with which real-time image analysis during the EOR operation alerts operators about any anomalies that occur.

**Figure 16:**
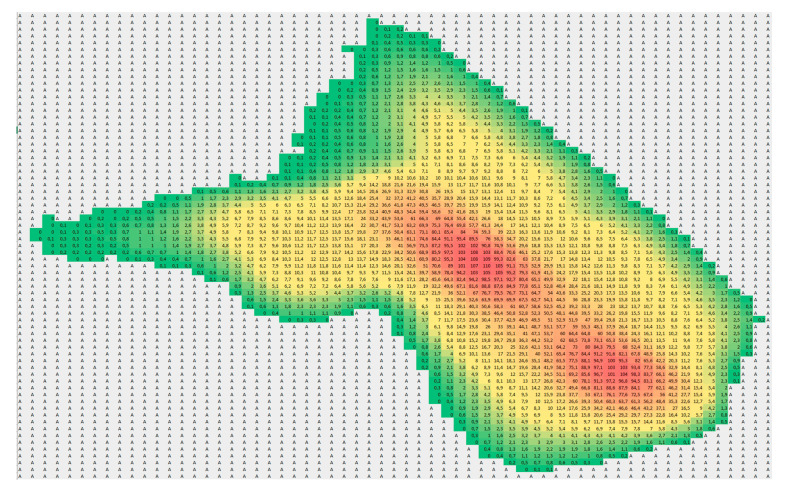
The result of the detection algorithm that normalised the resistance heater thermogram.

EOR track heaters marked as 1 in Figure 9, after correcting the span and temperature level (normalization), were presented to the operator and the analytics software. The proposed innovative detection algorithm for monitoring railway infrastructure at the outset rejects the irrelevant data for the assessment method and reduces the resolution of the image. Figure 16 shows the result of the detection algorithm which normalizes the heater thermogram. Gray cells (marked as A) indicate the interference area. The algorithm will not analyse this area because it is irrelevant for the assessment of the EOR system. The analysed areas are marked with colours from the RGB colour palette, conventionally shifting from green to red. The apparent temperature values for individual polygons of the thermogram are also mapped in these areas.

The red areas correspond to the operating heater. The visible gap in the red area reveals the element fixing the heater to the rail (pressure handle). The algorithm detects such areas concerning the known geometry of the track and turnout. Based on the image in the visible range and the thermogram, the turnout, resistance heaters, and damage are identified. The multi-sensor recognition allows for the identification of features of the object (Table 4). In the case of EOR system malfunctions, the colour gradient will be unsettled. Particular areas will be classified into the background area and extracted as disturbances.

## 5. Information System Supporting Thermographic Inspection

### 5.1. System Overview

Based on the data defined in Section 3 and Section 4, proprietary software (Python) was developed to analyse and predict the malfunction of EOR devices using thermal imaging and to potentially apply machine learning for image analysis.

**Figure 17:**
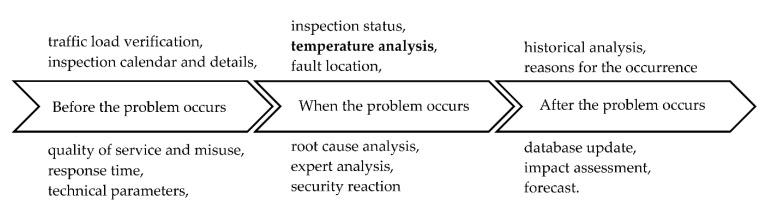
Data collection during the life cycle of the EOR heater (source: authors).

The program database contains the vocabularies and software components that enable the work at the test bench. A scheme for data collection and testing of EOR device components is shown in Figure 17. In order to correctly analyse the device, it is necessary to feed the database with the following data:

- Schematic representation of the test object for the recognition of the EOR elements placement;
- Operating temperature range, Table 2;
- Colour palettes e.g., rain, grey, iron;
- Specific operating parameters (temperature), Table 2;
- Interference parameters (temperature);
- Temperature below the values defined in Table 2;
- Symptoms of heater malfunction;
- Set of historic thermograms.

The database stores information on the actual condition and state of particular items, e.g., identifier, data on the type of heater, technical parameters, calendar of inspections, noted malfunctions and types of malfunctions, place of installation, local conditions, owner, etc. A separate database contains the historical thermograms with the manual inspection results as comparative material for machine learning.

**Figure 18:**
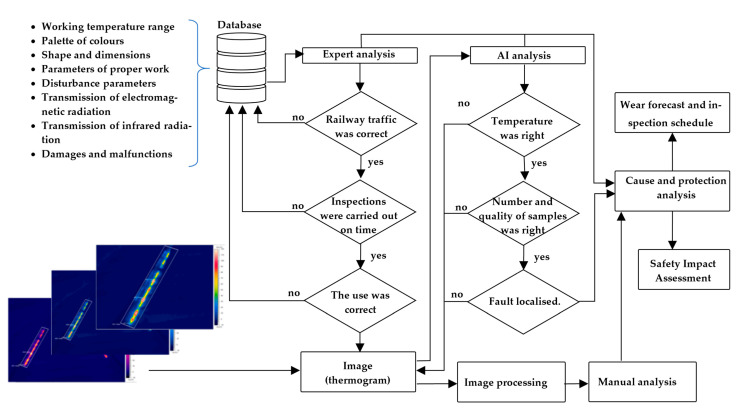
Algorithm of the method.

The algorithm of the proposed solution is shown in Figure 18.

The data range analysis system consists of the following components:

- Manual analysis module;
- Expert analysis module;
- Artificial intelligence module (SVM);
- Cause and safety analysis module.

The manual analysis module contains a set of tools processing the thermogram for direct visual inspection by the operator. The automatic adjustment requires trimming, selecting the colour pallet, zooming, and selecting the appropriate temperature span for proper visualization.

The expert analysis module allows information to be gathered on the object under study in its possible technical states. The proposed system can use the data related to local mechanical damage or the heater loop (heater jacket—dented, protruded, cut), mechanical damage of the supply cable, unsealing of the hermetic coupling connecting the supply cable with the heater bar, and number and frequency of inspections to speed up manual inspection or machine learning.

The artificial intelligence module allows thermal imaging inspection using ML techniques to be performed. The challenge is that the module does not yet utilize any pure mathematical models; it only utilizes data and probably expert estimates of quality, which are difficult to establish. Regardless of the list of relevant features provided by experts and ranges of measured physical quantities, these estimates may be contradictory or may contain errors, so it is important to use algorithms to obtain an objective assessment of the technical condition of EOR device components. Such an assessment may be carried out using machine learning techniques.

### 5.2. The Concept of Machine Learning Applied to Analysis of Thermograms for Fault and Damage Inspection

Machine learning is considered an effective tool for detecting anomalies based on pattern or image analysis. Yuan et al. [19] present an example of rail fastener clip damage detection by artificial neural network realization. In this study the single element is analysed for damage, and the neural network is proved to be a quick and reliable method for that. Neural networks can be used in any situation where a dependency or a batch of dependencies exists between the variables. The entanglement between variables can be complex and inexpressible in a classic way; however, it can be reflected by a neural network through correlations or differences between groups of cases. Neural networks can continuously monitor the condition of devices (like continuous sound analysis) or analyse waveforms of characteristics or images. The learned network will signal the need for inspection before a failure or an accident occurs.

Image analyse is an important application in the field of artificial intelligence [37] and analysis of thermograms is within the range of image classification problems (as analysed in [38]). There are studies proving the effectiveness of convolutional neural networks in image processing (like [43]), or other approaches used in thermograph analysis like [38,44,45]). Thermograms are images with a quite specific structure based on a standard colour scale. After proper orientation in space, it is relatively simple to extract the image of a heater, rail, or other element of a turnout and process the colour scale into its linear characteristics (if the transverse structure of the image is not considered). Extracting image features is the critical part of the pattern recognition. The quality of the extracted image affects the final recognition result.

The thermograms usually create small-scale data sets which are more susceptible to traditional machine learning models (as proved in [38]). A support vector machine (SVM) is proposed as a classification model in machine learning and data mining method for thermogram analysis [41]. SVM is mainly applicable to linearly separable data and linearly inseparable data. The kernel function technique is used in cases of linearly separable data. Radial basis kernel functions and polynomial kernel functions are widely used in SVM because of their classification capabilities [38].

The concept of machine learning applied to analysis of thermograms for fault and damage inspection involves: 1. Development of SVM for thermographic data from EOR imaging; 2. Development of thermographic images (filtering, scaling, trimming, simplification); 3. Preparation of SVM (learning) on historical data; 4. Analysis of thermographic images; and 5. Increasing the historical database, retraining.

## 6. Conclusions

The article analyses EOR devices for electric turnout heating as well as their operating states and ways of inspecting of them. A new method for identifying malfunctions and hazards in EOR operation by thermal imaging analysis is proposed. Thermal analysis can be used in manual mode as an insight into the device’s state or in automatic mode with a machine learning algorithm.

The share of tests, measurements, and thermal imaging in the exploitation processes concerning the use of EOR devices for electric heating of turnouts is increasing. In the railway industry, this technology is also increasingly used by infrastructure administrators to assess the broadly understood technical condition of equipment in which thermal processes take place. Observation and monitoring of these processes, also with the use of IT tools, according to the authors, is not the future but the necessary present, as a result of the implementation of new technologies. Visual inspection and thermal imaging measurements in the process of operating electrical and power and control equipment are becoming a standard. Technologies associated with thermal imaging are safe for the operator and allow contactless direct measurement of temperature on the surface of the object under study. The result of this measurement, when properly interpreted, allows operators to decide whether the device’s subassembly is efficient and whether it works or needs to be replaced or repaired. Interpretation of a device’s technical condition should be objective; this objectivity is provided by properly designed and properly used IT tools. Thermograms that are analysed by an operator are burdened with the operator’s subjective assessment, which is affected by knowledge of the device tested, knowledge of the processes occurring in the device tested, as well as knowledge of the ability of the device to operate at its stage of measurement, all of which is based on the operator’s interpretation of the measurement result. It is important to use the appropriate tools defined during the measurement and implemented by the thermal imaging camera and software at the stage of analysis and during interpretation of the result.

The results indicate that the thermal image analysis for a given area of track infrastructure can be an effective tool for damage and malfunction detection, especially when implemented as a tailored IT tool. The location of the defect will be revealed by an unevenly distributed thermal image or a lack of marked temperatures. The proposed system can support the estimation of wear time and the need for replacement of a given component. It can be unequivocally stated that thermograms with temperature distribution will allow the use of artificial intelligence for image analysis and threat identification.

The study conducted gives significant reasons to continue the process of expanding the area of infrastructure covered by the proposed system. Thermal imaging tools and their application to railroad infrastructure can significantly increase the level of safety by providing faster diagnosis of damaged elements and indications of emergency conditions. Covering other devices with the aforementioned monitoring will allow a comprehensive approach to railway infrastructure management through the simultaneous monitoring of a number of devices that influence the safety of passengers or goods that are transported by rail.

The presented results suggest that in transitional weather conditions in the studied latitude, the application of a system using thermal imaging techniques that monitors the wear of the many objects that support railway traffic will have a significant impact on reducing the number of disturbances in that railway traffic.

In the next stages of the research, the authors will address the problem of machine learning that is used for the recognition of thermographic images of EOR devices and the problem of the realization inspections in real time with the use of railway vehicles and a mobile version of the system.

## Figures and Tables

**Table 1:** Topics that arise in a literature review on railway elements monitoring.

Topics	Image Analysis	Energetic Efficiency	Diagnostics	IT Systems
General research/ technical requirements	[16,17,18,19,20,26,28]	[20,21,22]	[1,2,3,4,5,6,11,14,15,25]	[7,8,9,10]
System components/ damage	[24]	[12,13,14,37,38]	[25,29,30,39]	[23,40]
Modelling		[20,21,27]	[31]	[41,42]
Machine learning	[34,35]		[33]	[32,36,43,44,45,46]

**Table 2:** Dimensions and wattages of resistor heaters [30].

No.	Supply Voltage (V)	Nominal Rated Power (W)	Maximum Rated Power (W)	Minimum Rated Power (W)	Length L (mm)	Average Temperature with Heat Recovery	Steady-State Temperature of the Heater U = 253 V
1.	230	900	945	855	2800 ± 85	>160 °C	<400 °C
2.	230	1050	1102.5	997.5	3300 ± 100	>160 °C	<400 °C
3.	230	1250	1312.5	1187.5	3800 ± 115	>160 °C	<400 °C
4.	230	1600	1680	1520	4800 ± 145	>160 °C	<400 °C
5.	230	900	945	855	2800 ± 85	>160 °C	<400 °C

**Table 3:** Operational measurements for EOR devices—periodic inspections, thermovision inspection (own study based on [29].

No.	Name of the Device: Measuring Voltage	Type of Measurement	Technical Requirements	Frequency of Performance
1.	Systems with rated voltage up to 1 kV, weather robots, and control panels in metal housings Up = 1000 V	1. insulation resistance measurement	(a) R ≥ 1000 Ω/1 V for systems constructed before 07.08.1994	s
		2. Checking the operation of the anti-shock protection measures	(b) R ≥ 0.5 M Ω for systems constructed (upgraded) from 08.08.1994	Once every 5 years
2.	EOR switchboard: Up = 500 V	1. Checking the operation of the anti-shock protection measures	In accordance with the regulations on protection against electric shock	Once a year
		2. Measurement of insulation resistance of switchgear to reference earth	R > 2 M Ω	Once every 5 years
		3. Measuring the resistance between the metal casing and the reference earth	R > 20 Ω—for direct rail connection	
			R ≤ 20 Ω—for open rail connection	
		4. Measurement of earthing (protective earthing, working earthing of distribution point)	In accordance with the regulations on protection against electric shock	
		5. Thermovision inspection	Active (operational) EOR system, thermograms of switch cabinet equipment	Once a year
3.	EOR Transformer: - rectangular core Up = 500 V - toroidal core Up = 2.5 kV	1. Measurement of the insulation resistance between the live parts (primary and secondary windings)	R >5 M Ω	Once a year
		2. Insulation resistance measurement between live parts and the power transformer box casing (applies to metal casings only)		
4.	Heaters for heating: - resistors, - setting closures, - crossings with movable points Up = 500 V	1. Resistance measurement	In accordance with Table 8 in [29]	Before development
		2. Insulation resistance measurement between joined supply wires heater and heater jacket	R > 2 M Ω	
		3. Measurement of resistance between the protection wire and the heater jacket	R\le 0.1 Ω	
		4. Thermovision inspection	Active (operational) EOR system, radiator thermograms	After installation and once a year
5.	Power and control cables Up = 2.5 kV	Insulation resistance measurement	In accordance with attachments nos. 10, 11, 12 [29]	Once every 5 years
6.	Turnout, area of effective influence of EOR heaters	Thermovision inspection	Active (operational) EOR system, radiator thermograms	Once a year and after: (a) Installation, (b) Maintenance

**Table 4:** Object features in multi-sensor recognition. Own work on the basis of [40].

Sensor Type	Format Form	Characteristics
Thermal Imaging	2-dimensional image of heat emission	Shape (perimeter/field, aspect ratio, geometric moments) Texture, emission max/min, number and distribution of hot spots, thermal contrast
TV	2-dimensional image	Shape, texture, internal structure, contrast
